# Project BETTER: Preliminary Feasibility and Acceptability of a Technology-Delivered Educational Program for Pregnant and Postpartum People with Opioid Use Disorder

**DOI:** 10.1089/whr.2022.0046

**Published:** 2022-10-20

**Authors:** Sarah Martin, Anna Beth Parlier-Ahmad, Michelle Eglovitch, Steven J. Ondersma, Dace S. Svikis, Caitlin E. Martin

**Affiliations:** ^1^ School of Medicine, Virginia Commonwealth University, Richmond, Virginia, USA.; ^2^ Department of Psychology, Virginia Commonwealth University, Richmond, Virginia, USA.; ^3^ Division of Public Health and Department of Obstetrics, Gynecology, and Reproductive Biology, Michigan State University, East Lansing, Michigan, USA.; ^4^ Institute for Drug and Alcohol Studies, Virginia Commonwealth University, Richmond, Virginia, USA.; ^5^ Department of Obstetrics and Gynecology, School of Medicine, Virginia Commonwealth University, Richmond, Virginia, USA.

**Keywords:** acceptability, feasibility, opioid use disorder, postpartum, pregnancy, technology-based intervention

## Abstract

**Background:**

Postpartum people with opioid use disorder (OUD) report feeling underprepared for the pregnancy to postpartum transition. We developed a novel, technology-delivered educational intervention for pregnant and parenting people with OUD to address this gap. This study provides a theoretically grounded assessment of the feasibility and acceptability of a new technology-delivered educational intervention (Project BETTER) for pregnant and parenting people receiving medication for OUD (MOUD).

**Materials and Methods:**

Pregnant and postpartum people receiving MOUD were recruited from a perinatal addiction clinic research registry to pilot test the technology-delivered intervention. Participants completed one of three modules (Postpartum Transition, Neonatal Opioid Withdrawal Syndrome, or Child Welfare Interactions) and a survey assessing acceptability based on the theoretical framework of acceptability (TFA). We measured feasibility using process, resource, management, and scientific assessments. Demographics were self-reported. Clinical characteristics were abstracted from the medical record.

**Results:**

Feasibility was promising, with 17 of 28 participants approached (61%) agreeing to participate; 70% of these participants (*N* = 12; 58% White and 23% Black, all with public insurance) completed an intervention module and the study assessments, and all reported understanding how the modules worked. Acceptability was strong, with median ratings of 4 or 5 on a 5-point scale for all positively scored TFA domains. Compared to learning from a provider, participants also reported feeling more comfortable and less stigmatized learning from the intervention.

**Conclusion:**

Our theoretically grounded assessment suggests high feasibility and acceptability for Project BETTER, and provides justification for further evaluation in a clinical trial setting. Technology-delivered educational interventions may help reduce stigma and enhance prenatal education.

## Introduction

The rate of maternal opioid-related diagnosis at delivery hospitalization in the United States increased by 131% between 2010 and 2017.^1^ Consequently, pregnancy-associated mortality involving opioids is increasing in terms of both the rate and percentage of all pregnancy-associated deaths.^2^ Opioid overdose is a leading cause of pregnancy-associated death, with most deaths occurring in the postpartum period.^3^ According to a recent study of pregnant and parenting people in treatment for opioid use disorder (OUD), common contributors to overdose in the perinatal period are stress, lack of social support, infant care challenges, fear surrounding child welfare, and mental health issues.^4^ Infant diagnosis of neonatal opioid withdrawal syndrome (NOWS) at delivery may also be associated with increased risk of postpartum overdose.^5^

Overdose risk can be reduced by evidence-based treatment for OUD, including medication for OUD (MOUD), such as methadone and buprenorphine, and wrap-around services.^6^ However, birthing people with OUD are at a particularly increased risk for MOUD discontinuation and other compromised health outcomes after infant delivery,^7–10^ at least in part, due to unique postpartum stressors. Pregnant people report many challenges to OUD recovery during the transition from pregnancy to postpartum, including fear surrounding pain at labor and delivery, anxiety about NOWS, and interactions with child welfare, feelings of guilt, mood changes, parenting stress, and stigma.^11^ In addition, postpartum people with OUD report feeling underprepared and lacking prenatal education regarding expectations for the postpartum transition and care for infants who develop withdrawal symptoms.^12^

There is an urgent need for evidence-based, clinically feasible educational tools that can effectively equip the parent–infant dyad affected by OUD for continued recovery and optimal long-term outcomes.^13^ While NOWS education has been a component of broader parenting programs,^14^ only one study has reported on an educational intervention specific to NOWS for pregnant people in treatment for OUD.^15^ Although this study did not find a significant change in knowledge from baseline to post-education, participants indicated that the intervention was helpful in preparing them for the early postpartum period, while their newborns were hospitalized.^15^ More research is clearly needed to evaluate whether such programs can prove efficacious.

Technology-delivered interventions have demonstrated effectiveness as parental education tools in previous studies^16,17^ and may be more readily and cost-effectively implemented than person-delivered interventions.^18,19^ Interventions developed with the technology platform used in this study, the Computerized Intervention Authoring System (CIAS), version 3, have shown efficacy in peripartum populations for electronic screening and brief intervention, but such interventions have not, until now, been developed for education delivery.^20^

**FIG. 1.**
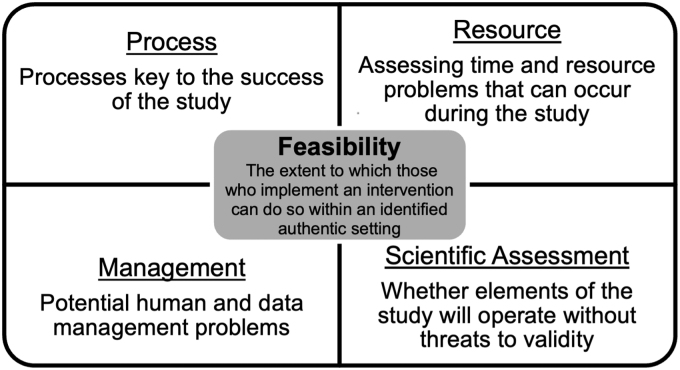
Typology of feasibility and pilot studies adapted from Tickle-Degnen.^23^

Patient educational interventions for this unique population must be both feasible to implement in clinical settings and acceptable to pregnant and parenting people in treatment for OUD.^11,15^ *Feasibility* is the extent to which those who implement an intervention can practically do so within an identified authentic setting.^21,22^ Tickle-Degnen adapted a comprehensive typology, originally used for drug trials, for feasibility measurement of pilot studies based on four key assessments: process, resources, management, and scientific basis (Fig. 1).^23^ *Acceptability* is historically a more ambiguous concept with definitions varying widely. Behavioral measures such as adherence and retention are often used as proxies for acceptability, with few studies using explicit measures.

**FIG. 2.**
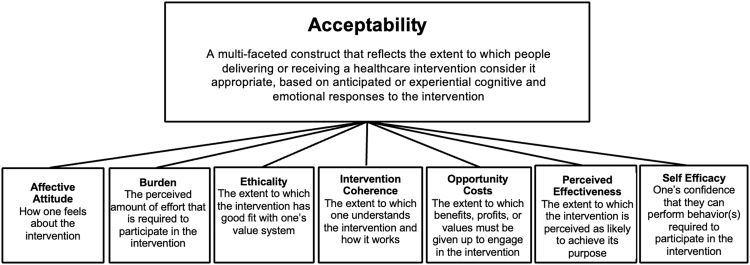
The theoretical framework of acceptability adapted from Sekhon et al.^25^

When patient-reported outcomes are used, they are typically limited to measures of satisfaction rather than comprehensive measures of acceptability.^20,24^ To address this gap, Sekhon et al. proposed the multiconstruct theoretical framework of acceptability (TFA) for health care interventions,^25^ which specifies seven specific domains seen as key in the evaluation of overall acceptability (Fig. 2). Lack of feasibility and acceptability can limit engagement and retention; therefore, comprehensive assessment of these concepts is a critical aspect of developing efficacious interventions.^26^

To our knowledge, there is no prior research evaluating technology-delivered prenatal educational interventions for birthing people receiving MOUD from a comprehensive feasibility and acceptability perspective. Thus, the primary objective of this study is to describe the preliminary feasibility and acceptability—in part, following the Tickle-Degnen^23^ and Sekhon et al.^25^ models—of using technology-delivered educational modules tailored for pregnant and postpartum people receiving MOUD. The goal of this study is to inform the next steps of evaluating the feasibility and acceptability of the modules as an intervention for this patient population using a clinical trial design.

## Materials and Methods

### Educational intervention: Project BETTER

All content for Project BETTER was developed using an iterative process with review from pregnant and parenting people receiving MOUD, as well as a multidisciplinary expert panel (see Parlier et al., 2022 under review [https://preprints.jmir.org/preprint/40162] for additional details).^27^ We used the CIAS, version 3.0^28^ (see www.cias.app for additional information), to develop three separate modules (pregnancy to postpartum transition, NOWS, and child welfare interactions) designed to take ∼20 minutes each to complete. Table 1 summarizes the content, delivery, and modes of access of the modules.

**Table 1.** Summary of Content, Delivery, and Accessibility of Project BETTER Educational Modules

Content	Postpartum transition	• Addiction recovery after pregnancy • Physical and emotional health after pregnancy
	NOWS	• What is NOWS, and how is it assessed and treated • Infant health promotion during and after pregnancy
	Child welfare interactions	• Child welfare referrals and child welfare case worker role • Expectations for interacting with child welfare
Delivery	Web-based modules led by an animated narrator with nonstigmatizing language, reflective listening and motivational interviewing techniques, interactive topic check-in questions, tailored content, and professionally produced educational videos featuring patient testimonies and health care providers trained in trauma-informed, person-centered care.	
Accessibility	Compatible with smart phone, tablet, or computer using a unique website link, accessible in the clinical space or remotely, and available indefinitely after completion.	

NOWS, neonatal opioid withdrawal syndrome.

### Study design

This study used both observational and survey data to assess feasibility and acceptability of novel technology-delivered, educational intervention modules for pregnant and postpartum people receiving MOUD. Participants who consented to participate in the study had 2 weeks to complete one of three educational modules in the Project BETTER intervention and a brief survey. Participants completed the module and survey either in the clinical space using a provided tablet or at home on their own electronic device. In addition, the research assistant completed a medical record review for each participant and recorded observational feasibility data. Participants received $20 compensation. All participants provided verbal consent, and this study was approved by the Institutional Review Board.

### Setting and participants

We recruited pregnant and postpartum people receiving MOUD treatment at a perinatal addiction clinic in a Medicaid-expanded state, which provides integrated prenatal, postpartum, and addiction care. The clinic provides care for all types of substance use disorders (SUDs) using a variety of treatment modalities, including medications and behavioral therapy. Addiction/OBGYN physicians and nurses provide educational content about the postpartum transition, NOWS, and child welfare over the course of pregnancy during prenatal visits using a trauma-informed and patient-centered approach. Participants were recruited from the clinic's research registry (*N* = 95). All perinatal addiction clinic patients are approached for participation in the research registry (85% recruitment rate).

Inclusion criteria included the following: at least 18 years of age, OUD diagnosis, currently receiving MOUD for at least 3 months, and have a historical pregnancy (within the last 12 months) or current pregnancy receiving MOUD. Of all registry participants, 46 participants were eligible for this study. Of those participants, 28 were contacted and 17 consented to the study and selected an intervention module based on their topic preference and remaining participant slots (4 available for each module). Our intended sample size for this preliminary study was 12 completers, with ∼4 participants completing each module. In total, *N* = 12 completed a module as well as the survey.

### Measures

#### Sociodemographic and clinical characteristics

Demographic and psychosocial data, including age, race, education level, health insurance status, household characteristics, psychiatric comorbidities, and overdose history, were self-reported by survey. Treatment and pregnancy characteristics were abstracted from participants' medical records.

We drew on previously published measures and theoretically grounded frameworks in the peer-reviewed literature to measure core components of feasibility and acceptability.

#### Feasibility

Based on the work by Tickle-Degnen (see Fig. 1),^23^ we measured feasibility using four types of assessments from observational and survey data. Process assessment (e.g., recruitment rate), resource assessment (e.g., availability of electronic devices to complete the intervention), and management assessment (e.g., number of protocol deviations) were evaluated with observational data. Scientific assessment (e.g., appropriateness of measures for the study population) was measured by the level of agreement with the following survey question: “I understand how the modules are supposed to work.”^29^ We report feasibility of evaluating the modules as an intervention in a randomized controlled trial (RCT) based on empirical and descriptive measures of these assessments.^23^

#### Acceptability

For this study, we created an 11-item self-report measure of acceptability (CARE questionnaire: Comprehensive Acceptability Review for E-interventions), corresponding to the seven component constructs of the TFA (see Fig. 2).^25^ Using a 5-point Likert scale, items within each of the TFA constructs for the self-report measure were adapted from previous studies using the TFA^29,30^ or CIAS^20^ and tailored to the target population (see Appendix Table A1 for acceptability measure).^25^ In addition, participants completed four study-specific acceptability questions.

### Data analysis

We generated descriptive statistics for sociodemographic and clinical characteristics of the study sample as well as for the feasibility and acceptability data (and their subcomponents) following the Tickel-Degnen and Sekhon models, respectively. Cronbach's alpha was calculated for items within each construct on the acceptability measure and was >0.7 for all acceptability subdomains. Analyses were performed using IBM SPSS Statistics 27.0.

## Results

### Characteristics of the study sample

As shown in Table 2, participants in this study were reproductive age (30.8 ± 4.1 years), White (58.3%), pregnant (33.3%) or postpartum (66.7%), and had a high school or equivalent level of education (66.7%). All had co-occurring psychiatric conditions (100%). Most were receiving buprenorphine (83.3%) and had been receiving MOUD for more than 2 years (66.7%).

**Table 2.** Descriptive Statistics of Pregnant and Parenting Participants in the Study Sample

Participant characteristics	*N* = 12, *N* (%)
Age, mean (SD)	30.8 (4.1)
Ethnicity	
Non-Latinx	12 (100)
Race	
American Indian or Alaska Native	1 (7.7)
Black	3 (23.1)
White	7 (58.3)
More than one race	1 (7.7)
Not reported	1 (7.7)
Education	
High school equivalent	8 (66.7)
>High school	4 (33.3)
Pregnancy status	
Pregnant	4 (33.3)
Postpartum	8 (66.7)
Self-reported psychiatric condition	12 (100)
Psychiatric medication	6 (50.0)
Past year behavioral health treatment	10 (83.3)
No. of prior pregnancies (Gravida), median (range)	3 (1–13)
No. of prior births (Parity), median (range)	2 (0–7)
No. of children in participant's custody, median (range)	1 (0–5)
Length of time in treatment receiving MOUD	
3–6 Months	1 (8.3)
6–12 Months	1 (8.3)
1–2 Years	2 (16.7)
>2 Years	8 (66.7)
Type of MOUD	
Buprenorphine	10 (83.3)
Methadone	2 (16.7)
Previous infant treated for NOWS	4 (33.3)
Previously worked with child welfare	6 (50.0)
Previous overdose	5 (41.7)
Medicaid health insurance	12 (100)
Intervention module selected	
Postpartum transition	4 (33.3)
NOWS	4 (33.3)
Child welfare	4 (33.3)

MOUD, medication for opioid use disorder; NOWS, neonatal opioid withdrawal syndrome; SD, standard deviation.

### Feasibility

**FIG. 3.**
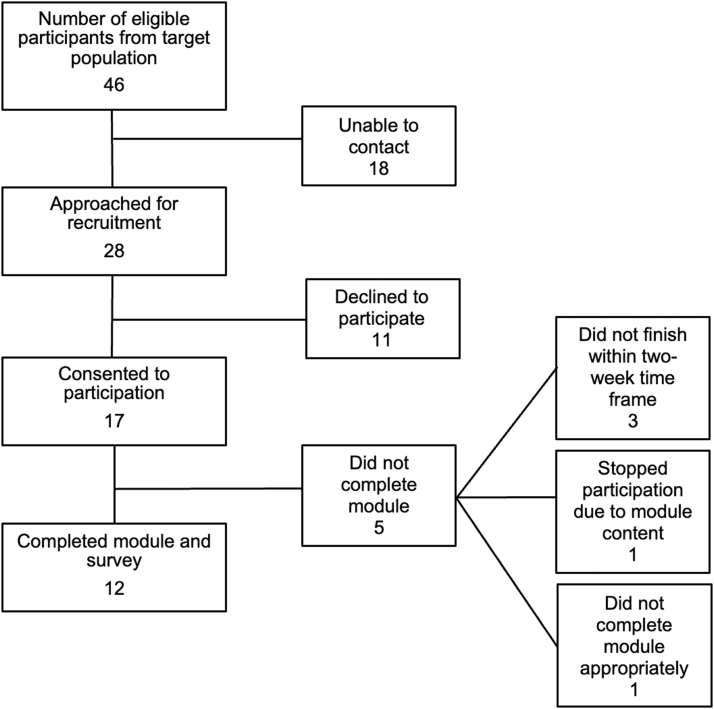
Feasibility^a^ process assessment flow chart based on the Tickle-Degnen model. ^a^Assesses the feasibility of processes that are key to the success of the study, including recruitment and participant completion of study procedures.

Participant flow can be seen in Figure 3, which shows a recruitment rate of 61% (i.e., 17 of 28 participants approached agreed to participate). Reasons cited for declining included lack of interest and lack of time. Three participants did not complete the intervention module within the required timeframe (2 weeks) and were lost to follow-up. One postpartum participant stopped the NOWS module prematurely noting it was difficult to complete, given her recent negative experiences with NOWS. She was provided counsel by a trained health professional. She declined further participation in the study. In addition, one participant disclosed that she did not actually complete the module; therefore, her responses were excluded from baseline characteristics and acceptability questionnaire data. Overall, 70% (*N* = 12) of recruited participants completed the intervention module.

All participants had the choice to complete study components in person or remotely, and 92% chose to complete the study remotely. Three participants reported technological difficulties, including having to restart the module if they needed to leave and return later (an issue that can be addressed by having participants create a CIAS login ID or use the same browser). In general, there was no significant human or data management issue, with only one instance of some missing survey data for the first participant. All participants reported that they understood how the modules were supposed to work (Table 3). Based on the 70% completion rate with few reported difficulties and positive responses to the feasibility question, intervention burden was low.

**Table 3.** Summary of Feasibility Data by Feasibility Component as Outlined by the Tickle-Degnen Model

Feasibility component^29^	Measure	Findings
Resources assessment	Time and resource problems	Three participants did not complete the intervention within the assigned 2-week time period One participant was initially unable to access CIAS
Management assessment	Human and data management problems	On three occasions, eligible participants were missed in clinic for recruitment Some missing demographic and acceptability data for one participant No protocol deviations No data management issue between REDCap and CIAS
Scientific assessment	Burdensomeness of intervention	100% of participants reported they understood how the modules were supposed to work

CIAS, Computerized Intervention Authoring System.

### Acceptability

Table 4 summarizes acceptability results. Overall, acceptability ratings were high across all constructs from the theoretical framework and the study-specific questions, suggesting high acceptability of intervention modules. The affective attitude of participants was very positive. In general, most participants liked the modules, with several participants expressing interest in completing additional modules. The individual intervention modules were not perceived as burdensome in terms of time or effort to those who completed them.

**Table 4.** Summary of Acceptability Data by Acceptability Constructs as Outlined by the Sekhon Model and Study-Specific Questions

Construct^25^	% Providing a positive rating of the intervention^a^	Median (interquartile range)^b^
Affective attitude: How an individual feels about taking part in an intervention		
Overall, how much did you like using the intervention?	66.7%	4 (3–5)
To what extent did you find the intervention to be interesting?	66.7%	4 (3–5)
Burden: The perceived amount of effort that is required to participate in the intervention		
Too much effort was required for me to participate^*^	75%	2 (1–2)^*^
Too much time was required for me to participate^*^	83.3%	2 (1–2)^*^
Ethicality: The extent to which the intervention has good fit with an individual's value system		
I felt like this was made for me and my community	75%	5 (4.25–5)
The intervention understood who I am and where I come from	75%	5 (4.25–5)
Intervention coherence: The extent to which the participant understands the intervention, and how the intervention works		
To what extent did you find the content of the intervention to be understandable?	100%	5 (5–5)
Opportunity costs: The extent to which benefits, profits, or values must be given up to engage in an intervention		
Was the time you spent completing the intervention worth it for the information you gained?	100%	5 (4–5)
Perceived effectiveness: The extent to which the intervention is perceived as likely to achieve its purpose		
If a friend were in need of similar help, how likely are you to recommend the intervention to them?	91.7%	5 (4–5)
Do you think that other people with your circumstances would be helped by this intervention?	100%	5 (4–5)
Self-efficacy: The participant's confidence that they can perform the behavior(s) required to participate in the intervention		
I am confident I completed the intervention in the way it is supposed to be done	100%	5 (4.25–5)
Study-specific adaptable questions		
Compared to receiving this information from your provider, how much did you like learning from the intervention?	75%	4 (3.25–4)
Compared to receiving this information from your provider, how comfortable did you feel learning from the intervention?	83.3%	4.5 (4–5)
I believe the intervention is effective for improving [my child's and my own] health and well-being	91.7%	5 (4.25–5)
Do you think other moms in treatment for OUD would be helped by this intervention?	100%	5 (4–5)

^*^ Indicates items that were reverse scored.

^a^ Percentage includes participants who provided a rating of 4 or 5 for most items; rating of 1 or 2 for reverse scored items. Response anchors varied (not at all, very much; or strongly disagree, strongly agree; etc.)

^b^ Item scores range from 1 to 5, with a higher score indicating greater agreement or a more positive impression of the intervention for most items; for reverse scored items, a lower score indicates greater agreement or a more positive impression of the intervention.

Furthermore, participants believed the intervention modules to have a good fit with their value systems, agreeing that the intervention was made for their community and understood where they come from. In terms of intervention coherence and perceived effectiveness, participants found the content of intervention modules to be understandable and believed that people with their circumstances would be helped by the intervention. Finally, participants believed that the time they spent completing the intervention modules was worth it for the information gained and felt confident that they completed the intervention modules as they were designed to be completed.

## Discussion

This study examined the preliminary feasibility and acceptability of Project BETTER, a novel, technology-delivered educational intervention for pregnant and postpartum people receiving MOUD. Results of this preliminary study suggest that Project BETTER is feasible for use in a clinical research setting and is likely highly acceptable to pregnant and postpartum people receiving MOUD. Overall, findings provide justification for the evaluation of the modules as an intervention in a subsequent clinical trial to further evaluate the intervention's feasibility and acceptability.

Feasibility is a challenging aspect of implementing interventions for populations in recovery from addiction due to the unique barriers and hardships they face.^31^ Based on our assessment of the processes, resources, management, and scientific processes, our findings demonstrate the feasibility of evaluating the modules as a technology-delivered educational intervention for pregnant and parenting people receiving MOUD in our planned RCT. Overall, our recruitment and study completion rates were similar to those reported in the literature for pregnant populations in SUD treatment and for behavioral interventions during pregnancy.^32–34^

One major factor that promoted feasibility of our intervention was the remote capability to complete all study components. In light of the COVID-19 pandemic, much of the clinical research world quickly adopted virtual methods. Early research from the pandemic suggests that remote options may foster research participation and reduce participant burden, increasing recruitment and study retention.^35^ Further investigation is needed to determine best practices for integrating virtual methods into studies along the translational research spectrum for populations commonly underrepresented in research.

Findings from this study represent the first theoretically grounded, comprehensive assessment of acceptability for technology-delivered interventions targeting SUD and its related behaviors. Prior research has established technology-delivered interventions as a promising mechanism to disseminate efficacious SUD treatment and recovery tools^19^; however, existing literature lacks comprehensive measures of their acceptability. As Sekhon et al.^25^ describe, acceptability is a necessary condition for intervention effectiveness. In our study, participants reported high acceptability ratings on the CARE Questionnaire, a novel survey measure grounded in strong theoretical foundation. We believe that this questionnaire could provide investigators with a useful tool to enhance the development and evaluation of technology-delivered interventions for individuals with SUD. For example, we intend to include the CARE Questionnaire in the follow-up assessments for our planned RCT evaluating Project BETTER.

Specifically, the ethicality construct of the TFA assessment—the extent to which the intervention has good fit with one's value system—may be particularly important for interventions tailored for individuals with SUD as an indicator of perceived stigma provoked by the intervention. Absence of stigma and bias is essential in interventions designed for people with SUD as perceived stigma is associated with compromised SUD outcomes, such as shorter retention and substance use recurrence.^36,37^

Technology-delivered educational content for birthing people with SUD may reduce the perceived stigma associated with SUD during pregnancy. Our participants reported that they preferred the module and were more comfortable learning from the module compared to their provider. This is consistent with previous research using CIAS in which most participants said that they preferred using the software over talking with medical staff about their substance use.^20^ More research is needed to assess the role of technology-delivered interventions in reducing stigma.

### Strengths and limitations

Our study has several limitations. First, the small sample size limits reliability and generalizability. Because of our small sample, we were only able to report aggregate feasibility and acceptability results, which may not reveal nuances about how participant and module characteristics impact feasibility and acceptability. Participants were engaged in a comprehensive care program for pregnant and parenting people with OUD at a large academic center that serves as a safety net for the region. Thus, our findings may not apply to populations with different clinical programs.

In addition, the data are impacted by selection bias as participants are recruited from a research registry, limiting our sample to those who are more agreeable to participation in research and are frequently further in their recovery and with more social stability. (Of note, the planned RCT will not be restricted to registry participants.) Despite these limitations, our study includes theoretically grounded assessments of feasibility and acceptability, differentiating it from previous work in the field and providing novel multidimensional feasibility and acceptability data.

## Conclusions

Findings demonstrate the feasibility and acceptability of tailored, technology-delivered educational intervention modules for pregnant and postpartum people receiving MOUD and provide justification for further evaluation of their feasibility and acceptability as an intervention in a clinical trial setting.

## Abbreviations Used

CIAS: Computerized Intervention Authoring System
OUD: opioid use disorder
MOUD: medication for OUD
NOWS: neonatal opioid withdrawal syndrome
SUD: substance use disorder
TFA: theoretical framework of acceptability

## Appendix

**Appendix Table A1.** Comprehensive Acceptability Review for E-Interventions (CARE Questionnaire)^25^

Construct^a^ and survey items	Item answer choices
Affective attitude	
Overall, how much did you like using the intervention?	Not at all (1); (2); (3); (4); Very much (5)
To what extent did you find the intervention to be interesting?	
Burden	
Too much effort was required for me to participate.^a^	Strongly disagree (1); Disagree (2); Neutral (3); Agree (4); Strongly agree (5)
Too much time was required for me to participate.^a^	
Ethicality	
I felt like this was made for me and my community.	Strongly disagree (1); Disagree (2); Neutral (3); Agree (4); Strongly agree (5)
The intervention understood who I am and where I come from.	
Intervention coherence	
To what extent did you find the content of the intervention to be understandable?	Not at all (1); (2); (3); (4); Very much (5)
Opportunity costs	
Was the time you spent completing the intervention worth it for the information you gained?	Not at all (1); (2); (3); (4); Very much (5)
Perceived effectiveness	
If a friend were in need of similar help, how likely are you to recommend the intervention to them?	Not at all (1); (2); (3); (4); Very much (5)
Do you think that other people with your circumstances would be helped by this intervention?	
Self-efficacy	
I am confident I completed the intervention in the way it is supposed to be done.	Strongly disagree (1); Disagree (2); Neutral (3); Agree (4); Strongly agree (5)

^a^ Indicates items which are reverse scored.
